# Intelligent Forging Driven by Mechanism–Data–Knowledge Fusion: A Review

**DOI:** 10.3390/ma19132737

**Published:** 2026-06-26

**Authors:** Haitao Wang, Guozheng Quan, Yichou Lin, Lin Gao, Yuqing Zhang, Xiao Liu, Haopeng Shi

**Affiliations:** 1 Chongqing Key Laboratory of Advanced Mold Intelligent Manufacturing, School of Materials Science and Engineering, Chongqing University, Chongqing 400044, China; 2 School of Materials Science and Engineering, Jiamusi University, Jiamusi 154007, China; 3 School of Mechanical and Electric Engineering, Sanming University, Sanming 365004, China; 4 Luoyang Zhongzhong Casting and Forging Co., Ltd., Luoyang 471039, China; 5 China National Erzhong Group Co., Ltd., Deyang 618000, China

**Keywords:** intelligent forging, process–structure–property relationship, mechanism–data–knowledge fusion, digital twin, quality prediction, adaptive control, intelligent manufacturing

## Abstract

Forging is a key manufacturing route for high-performance structural components, but its process design, quality prediction, and adaptive control still rely heavily on empirical rules, offline simulations, and fragmented production data. This review examines intelligent forging from the perspective of mechanism–data–knowledge fusion, with emphasis on forging-specific process chains, real alloy systems, model validation, and industrial maturity. To improve methodological traceability, a structured literature search was conducted using Web of Science Core Collection, Scopus, ScienceDirect, SpringerLink, and Google Scholar, covering studies published from 1996 to 2026. The screened literature was organized around process perception, mechanism-based modeling, data-driven learning, hybrid modeling, knowledge representation, digital twins, online prediction, and adaptive regulation. Representative cases are discussed for closed-die forging, open-die/large forging, multistage forging, radial forging, and forging of aluminum alloys, titanium alloys, steels, and Ni-based superalloys. Particular attention is given to how specific models are validated, including independent experiments, finite-element benchmarks, industrial datasets, new geometries, sensor noise, and cross-material or cross-equipment transfer. The review further distinguishes consolidated technologies, such as FEM-based process simulation and die/preform optimization, from methods still under validation, including hybrid digital twins, sensor-updated models, and adaptive control. Large-model-assisted forging is considered a prospective direction mainly for information retrieval, case recovery, diagnostic support, and engineer-supervised recommendation rather than unsupervised real-time control. This review provides a more process-specific and critically assessed reference for developing explainable, validated, and deployable intelligent forging systems.

## 1. Introduction

Forging is one of the most important near-net-shape manufacturing routes for producing high-performance structural components with superior strength, toughness, fatigue resistance, and structural reliability. Owing to its capability to refine cast microstructures, reduce internal defects, and establish favorable flow-line distributions, forging has long been indispensable in aerospace, transportation, energy, and heavy-duty equipment manufacturing, particularly for wrought aluminum and other high-performance alloys [1,2,3,4,5,6,7]. At the same time, the continuing demand for lightweight structures, high service reliability, reduced manufacturing cost, and stable batch quality has driven forged components toward larger dimensions, more complex geometries, tighter tolerances, and increasingly stringent requirements for microstructural homogeneity and property consistency [1,2,7].

**Figure 1:**
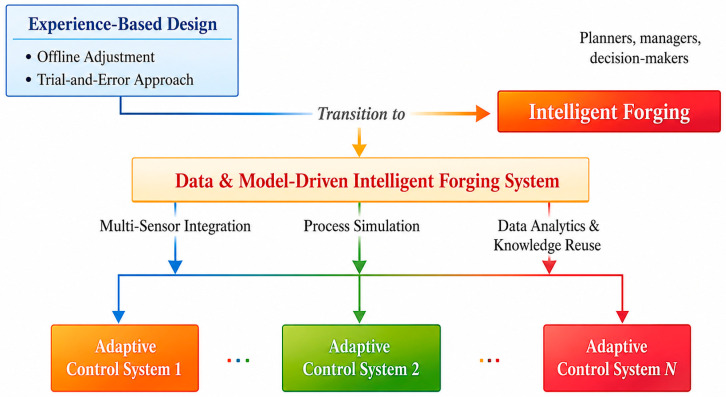
Transition from conventional trial-and-error forging development to intelligent forging driven by data, models, and knowledge. The ellipses indicate extensibility to multiple adaptive control systems. Arrows indicate the transition and information flow, and colors distinguish the main functional modules.

Although computer-aided engineering has significantly improved forging process development, industrial practice still relies heavily on empirical knowledge, offline finite-element simulation, and repeated trial-and-error [2,3,4,5,6,7,8,9,10,11,12,13,14,15]. This challenge is particularly pronounced in multistage and large-scale forging, where final quality is governed by the strong coupling of temperature, strain, strain rate, friction, die deformation, and microstructural evolution, resulting in a highly nonlinear and strongly path-dependent process [2,3,4,5,6,7,8,9,10,11,12,13,14,15]. Consequently, critical objectives, including die filling, defect suppression, grain refinement, anisotropy control, dimensional accuracy, and tool-life improvement, are difficult to optimize simultaneously. Moreover, data generated throughout billet preparation, heating, preforming, finish forging, heat treatment, inspection, and machining are often fragmented and weakly connected, thereby limiting knowledge reuse and hindering effective closed-loop control. As shown in Figure 1, this situation reflects a broader transition from conventional forging development, dominated by experience-based design and offline adjustment, to intelligent forging, which emphasizes the integration of multi-source sensing, process simulation, data analytics, knowledge reuse, and adaptive decision-making. Therefore, intelligent forging should be regarded not simply as a technological upgrade of individual tools, but as a systematic transformation of the entire forging workflow toward data-enabled and model-supported control.

Against this background, recent advances in intelligent manufacturing offer a promising pathway for transforming forging from an experience-dominated process into an adaptive and knowledge-enabled manufacturing system. Machine learning has demonstrated considerable potential in prediction, diagnosis, and optimization, whereas physics-informed learning has further highlighted the value of integrating governing laws with data-driven models to improve robustness, interpretability, and transferability [16,17,18,19]. In parallel, digital twins have emerged as a practical framework for real-time state mapping, predictive analysis, and decision support in manufacturing systems [17,18,20,21,22,23,24]. Knowledge-graph-based approaches have broadened the scope of intelligent process planning by enabling structured representation and reasoning over heterogeneous process knowledge [25,26,27]. Meanwhile, human-in-the-loop and Operator 5.0 concepts have underscored the continuing importance of engineering expertise in future intelligent production environments [28,29].

Literature search strategy. To make the review methodology more transparent, the literature was searched in Web of Science Core Collection, Scopus, ScienceDirect, SpringerLink, and Google Scholar for the period 1996–2026. The main search combinations included “forging AND intelligent manufacturing”, “forging AND machine learning”, “forging AND digital twin”, “forging AND hybrid model”, “forging AND knowledge graph”, “forging AND preform optimization”, “forging AND microstructure prediction”, and “forging AND closed-loop control”. Studies were included when they addressed forging or closely related metal-forming processes and provided clear information on process route, alloy or material system, model type, data source, validation method, or deployment scenario. Studies were excluded when they were only general AI-manufacturing discussions, lacked a forging-relevant application context, provided no methodological or validation information, or duplicated information already covered by a more complete source.

Representative advances in intelligent forging are summarized in Table 1 according to their technical functions and application scenarios, rather than by geographical origin. This neutral organization highlights complementary progress in digital process design, process–performance mapping, intelligent thermal control, quality traceability, multi-field modeling, process data acquisition, cloud-control platforms, and closed-loop optimization. It therefore avoids geographically based categorization while maintaining a balanced overview of the field.

At the enabling-technology level, prior studies have established machine learning, digital twins, knowledge graphs, visual analytics, and human-centered control as important foundations for intelligent manufacturing [16,17,18,20,21,22,25,26,28,29]. These works provide methods for prediction, diagnosis, real-time state mapping, semantic organization, and human-supervised decision support, all of which are relevant to the transition from empirical forging practice to model-supported and knowledge-enabled manufacturing.

Forging-specific studies have further translated these enabling technologies into process design, die/preform optimization, and microstructure-sensitive regulation. Examples include deformation-route studies that connect strain accumulation with microstructure and mechanical properties [5,7], finite-element and neural-network-assisted die design [30,31,32,33], and physically based models for microstructure evolution during hot working and cogging [34,35]. This functional presentation emphasizes methodological complementarity and avoids presenting research contributions as separate regional categories.

**Figure 2:**
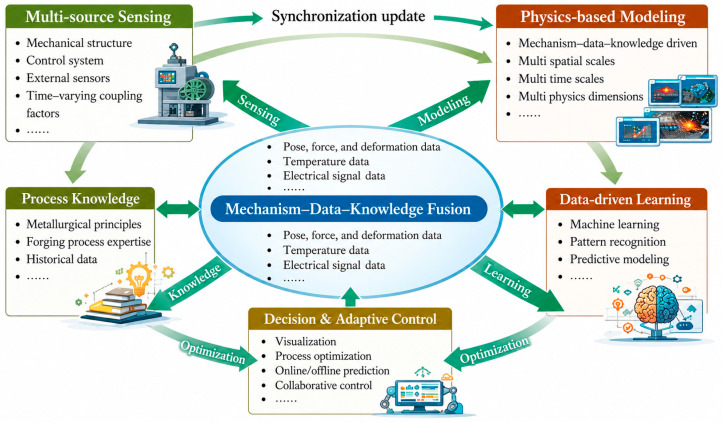
Mechanism–data–knowledge fusion framework for intelligent forging across sensing, modeling, optimization, and adaptive control, incorporating the closed-loop interaction among sensing, modeling, optimization, and regulation. Arrows indicate the closed-loop flow of data, knowledge, modeling, and control, and colors distinguish the principal functional modules.

Despite recent progress, the application of intelligent manufacturing methodologies to forging remains incomplete. Existing studies still tend to focus on a single algorithm, process stage, or data source, whereas industrial forging is inherently a full-chain process involving billet preparation, heating, preforming, finish forging, heat treatment, inspection, machining, and feedback regulation. In forged components, local thermal history and deformation path determine not only shape formation and defect occurrence, but also grain refinement, recrystallization behavior, texture evolution, precipitation response, anisotropy, and ultimately service performance [1,2,3,4,5,6,7,8,9,10,11,12,13,14,15,32,33,34,35]. Accordingly, intelligent forging should not be regarded as a simple process-parameter optimization problem, but rather as a multilevel modeling problem requiring the coordinated integration of physical mechanisms, production data, and engineering knowledge. As shown in Figure 2, this concept can be described by a mechanism–data–knowledge fusion framework, which links multi-source sensing, physics-based modeling, data-driven learning, process knowledge, and decision-making modules within a closed-loop architecture. The framework therefore reflects the transition from isolated process analysis toward integrated sensing, modeling, optimization, prediction, and adaptive control, and captures the central scientific and engineering logic of intelligent forging.

From this perspective, intelligent forging is better understood as a mechanism–data–knowledge fusion paradigm than as a straightforward extension of automation or digitalization. Mechanism models provide physical consistency, data models enable responsiveness to complex nonlinear behavior, and knowledge models ensure interpretability and continuity of engineering experience. Only through the effective fusion of these elements can forging evolve from offline empirical optimization toward explainable, adaptive, and trustworthy intelligent manufacturing. Accordingly, the present review aims to clarify the conceptual boundary of intelligent forging, summarize recent progress in modeling and intelligent control, identify the principal technological bottlenecks that still hinder practical implementation, and provide a structured reference for the future development of industrially deployable intelligent forging systems.

## 2. Framework of Intelligent Forging

### 2.1. Definition and Scope of Intelligent Forging

Intelligent forging can be defined as a forging-oriented manufacturing paradigm in which process perception, modeling, prediction, decision-making, and control are integrated through the coordinated use of physical models, production data, and engineering knowledge. Unlike conventional digital forging, which mainly depends on numerical simulation, process databases, or isolated automation units, intelligent forging aims to establish a closed-loop system capable of sensing process states, interpreting process–structure–property relationships, optimizing process parameters, and adaptively regulating production behavior under variable operating conditions [17,25,26,36,37,38]. Therefore, intelligent forging should be understood not simply as a digital upgrade of conventional forging, but as a transition from experience-based process development to model-assisted and knowledge-supported manufacturing.

To maintain consistent terminology, mechanism–data–knowledge fusion is used here to denote the coordinated integration of physical mechanisms, production data, and codified engineering knowledge. A hybrid model refers to a model that explicitly combines at least two of these sources, rather than a purely empirical algorithm. A digital twin refers to a dynamically updated virtual representation linked to a physical forging process or asset. Adaptive regulation denotes supervised process adjustment based on updated state information, whereas closed-loop control denotes the feedback architecture that connects sensing, prediction, decision-making, and execution.

**Figure 3:**
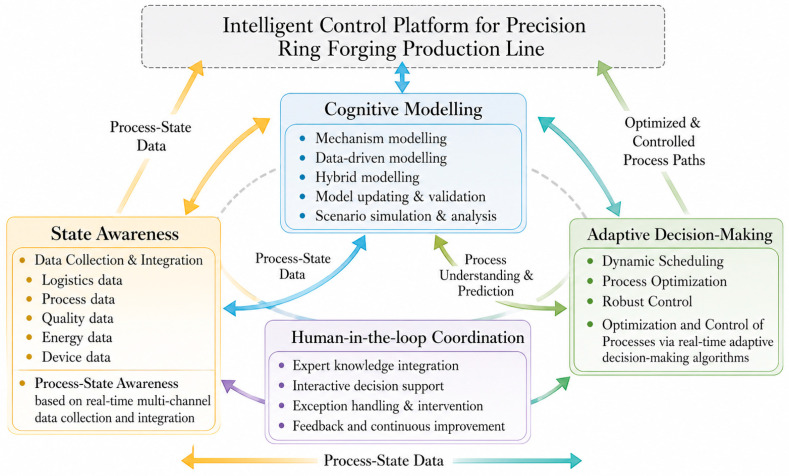
Conceptual framework of intelligent forging, highlighting state awareness, cognitive modeling, and adaptive decision-making. Arrows indicate information and control flow, and colors distinguish the principal functional modules.

From a functional perspective, intelligent forging is characterized by three closely related capabilities: state awareness, cognitive modeling, and adaptive decision-making. State awareness provides real-time information on temperature, force, displacement, deformation history, and quality responses throughout the forging process. Cognitive modeling establishes the relationships among process variables, deformation behavior, microstructural evolution, and final properties through mechanism-based, data-driven, or hybrid approaches [17,25,26,36,37]. Adaptive decision-making further uses this information to update process windows, optimize operating parameters, and support online regulation under changing production conditions [2,36,38]. As shown in Figure 3, these three capabilities form a progressive and closed-loop structure, in which process-state awareness provides the data foundation, cognitive modeling enables process understanding and prediction, and adaptive decision-making supports optimization and control.

The connotation of intelligent forging therefore extends beyond the simple introduction of artificial intelligence algorithms into forging practice. It requires the coordinated integration of process objectives, process routes, sensing systems, model architectures, knowledge organization, and control logic. In industrial applications, intelligent forging should support process understanding, quality prediction, multi-objective optimization, and adaptive regulation with full-chain traceability [2,17,25,26,36,37,38]. However, it does not imply fully autonomous production. For large, complex, or high-value forged components, expert judgment remains necessary for billet design, heating schedules, deformation paths, lubrication conditions, and defect-risk interpretation. For this reason, human-in-the-loop coordination remains an essential component of intelligent forging [28,29].

### 2.2. Process Chain and Major Quality Objectives

Forging should be understood as a full-chain manufacturing process rather than a single forming step. In closed-die and multistage forging, the chain typically includes billet preparation, heating, lubrication/descaling, preforming, finish forging, trimming, heat treatment, inspection, and machining. In open-die and large-forging operations, additional steps such as upsetting, drawing-out, cogging, mandrel drawing, intermediate reheating, controlled cooling, straightening, and defect-removal operations may be required. Radial forging has its own equipment-control logic, involving hammer motion, rotation/feeding, mandrel support, temperature evolution, and dimensional control. Since each stage affects thermal history, strain path, stress state, and microstructure, the final quality of a forged component is a cumulative result of full-chain interactions [2,3,9,24].

Within this process chain, the governing variables are distributed across several coupled domains. Thermal variables, such as billet and die temperatures, heating uniformity, heat transfer, and cooling history, strongly affect flow stress and microstructural evolution. Mechanical variables, including strain, strain rate, stress state, friction, die motion, and press characteristics, govern material flow, cavity filling, and defect formation. Material variables, such as initial microstructure, alloy composition, grain size, phase constitution, and precipitate state, determine hot workability and final properties. In addition, operational variables, including equipment condition, sensing reliability, and execution stability, influence process repeatability and manufacturing robustness [2,3,9,18,20,22,24]. Therefore, the process chain of intelligent forging should be viewed not simply as a sequence of operations, but as a dynamic coupling of thermal, mechanical, material, and control factors.

The major quality objectives of intelligent forging can be grouped into four categories: forming quality, microstructural quality, property quality, and manufacturing quality. Forming quality includes die filling, geometric integrity, dimensional accuracy, and defect suppression. Microstructural quality involves grain size, recrystallization, texture evolution, precipitate behavior, and structural homogeneity. Property quality covers strength, ductility, toughness, fatigue resistance, creep resistance, and service stability. Manufacturing quality includes process consistency, die life, energy consumption, production rhythm, traceability, and cost-effectiveness [2,3,9,24]. In practice, these objectives are strongly coupled and often mutually constrained. For example, improving cavity filling may increase local deformation and temperature rise, which can subsequently affect recrystallization behavior, die wear, and residual stress. Therefore, intelligent forging must address multi-objective coordination rather than single-index optimization.

**Figure 4:**
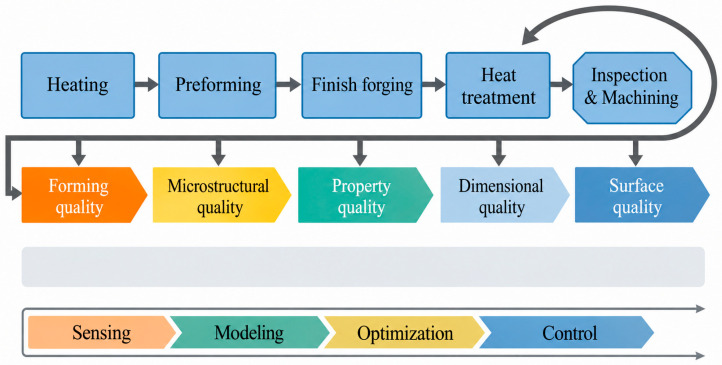
Typical process chain of intelligent forging and the associated major quality objectives across forming, microstructural, property, and manufacturing dimensions. Arrows indicate process progression and feedback, and colors distinguish process stages and quality-objective categories.

As shown in Figure 4, the process chain of intelligent forging is directly linked to its major quality objectives. Heating determines the initial process window and microstructural stability; preforming controls material distribution and folding risk; finish forging governs final geometry, local strain, and residual stress; heat treatment modifies grain structure, phase state, precipitation, and final properties; and inspection/machining provide the delayed quality responses needed for feedback learning. For large steel or superalloy forgings, the key issue is often through-thickness uniformity; for precision closed-die forgings, the key issue may be filling, dimensional accuracy, and die life; and for titanium or aluminum alloy forgings, the dominant concerns may include texture, recrystallization, and property anisotropy. This is why intelligent forging requires process-specific quality objectives rather than a universal optimization index.

### 2.3. Mechanism–Data–Knowledge Fusion Framework

The above discussion indicates that intelligent forging should be built on a mechanism–data–knowledge fusion framework. In this framework, mechanism refers to the physical principles and process models that describe deformation, heat transfer, microstructural evolution, damage, and equipment–process interactions. Data refers to the heterogeneous information obtained from simulations, sensors, production records, inspection results, and historical cases. Knowledge refers to codified engineering experience, process rules, design logic, quality heuristics, and structured relations among process entities [17,25,26,36,37]. The purpose of this framework is to integrate these three resources into a unified system for process understanding, quality prediction, optimization, and adaptive control.

Within this framework, the mechanism layer provides physical consistency and explanatory capability. It typically includes constitutive descriptions, finite-element simulation, recrystallization and grain-growth models, damage criteria, thermal boundary conditions, and process-equipment coupling models [2,3,9,24,38]. The data layer provides empirical adaptability and process responsiveness, including online sensing data, simulation datasets, inspection results, microstructure observations, property measurements, and anomaly records [17,18,20,22,36,37]. The knowledge layer provides semantic organization and decision continuity through process cards, expert rules, case libraries, design criteria, and knowledge graphs [25,26]. These three layers are complementary: mechanism models ensure physical plausibility, data enable real-time adaptation, and knowledge supports interpretability and engineering decision-making.

A complete fusion framework should therefore include five key modules: multi-source sensing, state representation, hybrid modeling, decision-making and optimization, and execution-feedback regulation. Multi-source sensing acquires process-state and quality-related information from the shop floor and associated digital systems. State representation transforms raw data into interpretable descriptors of thermal state, deformation history, equipment condition, and quality risk. Hybrid modeling combines mechanism-based simulation, data-driven learning, and knowledge reasoning to establish reliable mappings from process variables to geometry, microstructure, and performance. Decision-making and optimization determine or update process routes and operating windows under multiple objectives and constraints. Execution-feedback regulation then closes the loop by comparing predicted and actual responses and updating process parameters, model states, or knowledge bases accordingly [17,18,20,22,25,26,28,29,36,37,38].

Consistent with the closed-loop logic already integrated in Figure 2, the core value of mechanism–data–knowledge fusion lies in the interaction among sensing, modeling, optimization, and regulation. In this architecture, mechanism models constrain learning outcomes and improve physical consistency, data compensate for model simplifications and support real-time responsiveness, and knowledge provides semantic structure and actionable engineering logic [17,25,26,36,37]. Therefore, an additional schematic is unnecessary; the discussion is retained here in text form to avoid redundant illustration while emphasizing that intelligent forging is a dynamic manufacturing paradigm in which process physics, production data, and engineering knowledge are continuously coupled and updated.

## 3. Data and Modeling Foundations

### 3.1. Multi-Source Data in Forging Processes

The development of intelligent forging depends fundamentally on the availability, quality, connectivity, and semantic consistency of process data. Unlike conventional forging studies that may rely on isolated experiments or FEM outputs, intelligent forging requires multi-source data that describe process states, material responses, equipment conditions, and quality outcomes across the full manufacturing chain [16,17,24,36,39,40]. These data are heterogeneous: thermocouple and infrared measurements describe surface or furnace temperature; press systems record load, stroke, energy, and velocity; vision or laser systems provide surface or dimensional information; metallography and EBSD provide delayed microstructural labels; and mechanical testing provides sparse but decisive property labels. Therefore, the data problem in forging is not simply “large data” but the reliable alignment of incomplete online signals with sparse offline quality evidence.

**Figure 5:**
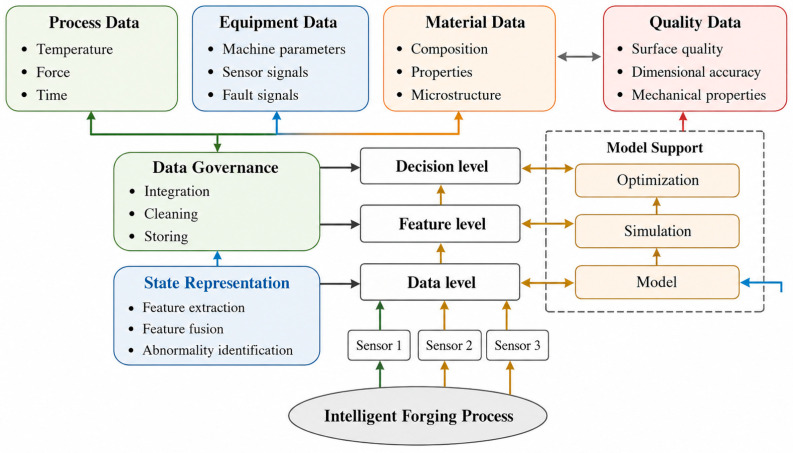
Multi-source data foundation in intelligent forging, showing the integration of process, equipment, material, and quality data into data governance, state representation, and model support. Arrows indicate data flow, and colors distinguish data sources and functional layers.

In general, forging-related data can be classified into four categories: process data, equipment data, material data, and quality data. Process data include temperature, force, stroke, deformation time, energy input, lubrication condition, and thermal history. Equipment data involve press status, die condition, machine compliance, maintenance records, and execution stability. Material data cover alloy composition, initial microstructure, constitutive behavior, and phase or precipitate states. Quality data include dimensions, geometric deviations, defect records, microstructural observations, mechanical properties, and residual stress or distortion responses [16,17,24,39,40]. As shown in Figure 5, these data sources should not be treated as isolated information streams, but as an integrated data foundation that supports data governance, state representation, and model input for subsequent prediction and control.

A major challenge is that forging data are unevenly distributed across the process chain. Some variables, such as press force, ram position, and furnace temperature, can be monitored continuously, whereas others, such as internal strain distribution, local microstructure, and bulk residual stress, remain difficult or costly to measure directly during production. In addition, online data are usually high-frequency but indirect, while offline data from microstructure characterization, property testing, or defect inspection are more explicit but sparse and delayed [17,24,39,40]. Therefore, multi-source data in forging should not be regarded simply as a large dataset, but rather as a layered information structure that requires synchronization, cleaning, alignment, and semantic interpretation before it can support intelligent modeling and control.

After proper processing, raw data from process monitoring, equipment operation, materials characterization, simulation, and inspection can be transformed into interpretable descriptors of thermal state, deformation history, process variability, and quality risk. In this sense, the multi-source data foundation is the starting point of intelligent forging, because it determines both the observability of process states and the reliability of subsequent modeling, optimization, and adaptive regulation.

### 3.2. Mechanism-Based, Data-Driven, and Hybrid Modeling Methods

Modeling is the core analytical layer of intelligent forging because it transforms raw process information into interpretable relationships among process variables, geometry, microstructure, and final properties. In general, the modeling methods used in forging can be classified into three categories: mechanism-based models, data-driven models, and hybrid models [17,24,25,36,39,40,41]. Although these approaches differ in modeling logic, data dependence, and application scope, they should be understood as complementary rather than competing methods.

Mechanism-based models are grounded in physical principles and metallurgical laws. They typically include constitutive descriptions, finite-element simulation, heat-transfer analysis, recrystallization and grain-growth models, damage criteria, and process-equipment coupling models. Their main advantage lies in providing physical consistency and explanatory capability, which makes them particularly important for understanding material flow, temperature evolution, defect formation, and microstructural development under varying process conditions [24,38,39]. However, they are often computationally expensive, sensitive to parameter calibration, and difficult to update in real time.

By contrast, data-driven models learn patterns directly from process records, sensing data, simulation datasets, or inspection results. Such models have been applied to defect identification, surrogate modeling, parameter optimization, state estimation, quality prediction, and process monitoring in forging and metal forming [16,17,36,39,41]. Their main strengths are modeling speed, adaptability, and efficiency in handling large datasets. However, purely data-driven approaches are often limited by data scarcity, inconsistent labeling, reduced interpretability, and weak transferability across different alloys, tools, and equipment conditions. These limitations are especially important in forging, where many local material states are difficult to observe directly.

**Figure 6:**
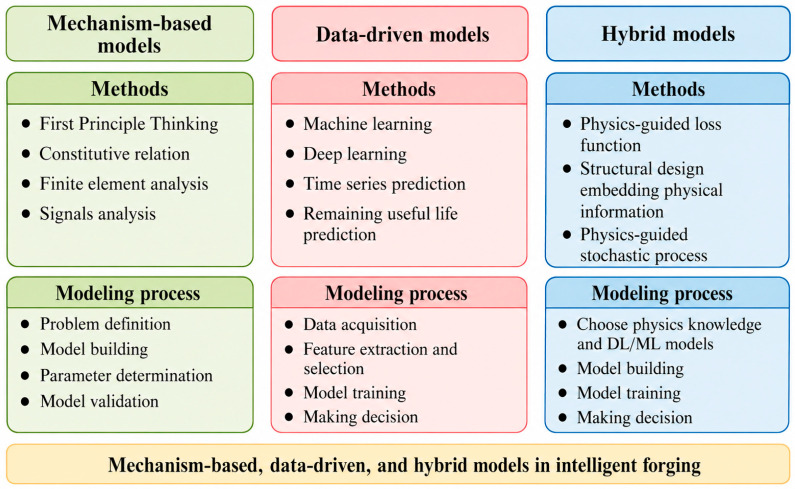
Major modeling paradigms in intelligent forging, including mechanism-based, data-driven, and hybrid models. Colors distinguish mechanism-based, data-driven, and hybrid modeling paradigms.

For this reason, hybrid modeling has become an increasingly important direction in intelligent forging. Hybrid models combine the physical consistency of mechanism-based simulation with the responsiveness of data-driven learning. Typical examples include simulation-informed surrogate models, reduced-order digital twins, physics-informed machine learning, and fusion frameworks in which sensing data are used to update process models or constrain predictions [17,24,36,40,41]. As shown in Figure 6, the relationship among the three modeling paradigms is essentially complementary: mechanism-based models emphasize physical consistency, data-driven models emphasize rapid response and pattern extraction, and hybrid models aim to integrate the strengths of both. Their common objective is to provide reliable predictions of geometry, defects, microstructure, properties, and optimized process windows.

For this reason, hybrid modeling has become a central direction in intelligent forging, but the term should not be used as a vague label. In forging, hybrid models can be divided into FEM-trained surrogates, physics-informed learning models, reduced-order digital twins, sensor-updated state-estimation models, and expert-rule-integrated models. These categories differ in data requirements, physical constraints, update mechanisms, and deployment targets. Their common purpose is to combine the physical plausibility of mechanism models with the speed, adaptability, and pattern-recognition ability of data-driven models.

To clarify how these hybrid strategies differ in practical forging applications, Table 2 summarizes their core principles, application scenarios, validation focuses, main limitations, and representative supporting references. This revision makes the classification traceable to specific studies rather than presenting it only as a conceptual grouping. The comparison indicates that FEM-trained surrogate models are suitable for rapid design screening, physics-informed learning is more appropriate for sparse-data microstructure/property prediction, reduced-order digital twins and sensor-updated models support online correction, and expert-rule-integrated models are useful for process planning and diagnosis.

Therefore, model selection in intelligent forging should be task-specific. If the objective is die filling or forming load, FEM-based simulation or FEM-trained surrogates are usually suitable. If the objective is grain size, recrystallized fraction, or property distribution, metallurgical models and physics-informed learning are more appropriate. If the objective is online correction, a sensor-updated reduced-order model or digital twin is required. If the objective is process planning or diagnosis, structured expert knowledge and case reasoning must be included. This task-oriented view makes the review more methodologically traceable and avoids treating all intelligent models as interchangeable algorithms.

### 3.3. Knowledge Representation and Digital Support

In addition to data and models, intelligent forging requires an explicit framework for representing and reusing engineering knowledge. This is essential because many forging decisions still depend not only on measurable variables, but also on process know-how accumulated through industrial practice, such as billet selection, preform design, heating strategy, defect diagnosis, and compensation rules. If such knowledge remains implicit, it is difficult to transfer, standardize, or integrate into intelligent decision-making systems [17,25,26,49]. Therefore, knowledge representation is a key foundation for linking process theory, data analysis, and engineering practice.

In intelligent forging, knowledge can be represented as process cards, rule sets, case libraries, ontologies, semantic relations, and knowledge graphs. The key point is that engineering knowledge must be represented in a form that is searchable, auditable, and computable [25,26,49]. Typical forging rules include alloy-dependent initial heating ranges, maximum reduction per pass, temperature thresholds for intermediate reheating, criteria for avoiding folds or laps, die-temperature and die-wear limits, lubricant selection according to alloy and temperature, allowable reheating times, dimensional compensation after cooling or machining, and alarm rules for abnormal load-stroke curves. Knowledge graphs are useful because they can connect materials, equipment, operations, parameters, defects, microstructures, properties, and inspection results in a unified semantic network.

Digital support provides the infrastructure through which represented knowledge can be applied in practice. In intelligent forging, such support may include digital twins, cloud platforms, traceability systems, historical databases, and integrated production-management environments [17,22,24,36,40]. These tools do not replace process knowledge; rather, they provide a computational environment in which knowledge can be linked with data streams, process models, and decision modules. For example, digital twins can integrate process-state information with reduced-order models and historical cases, while cloud platforms can support cross-stage data sharing, model updating, and process traceability. In this way, digital support makes process knowledge not only storable, but also computationally usable.

**Figure 7:**
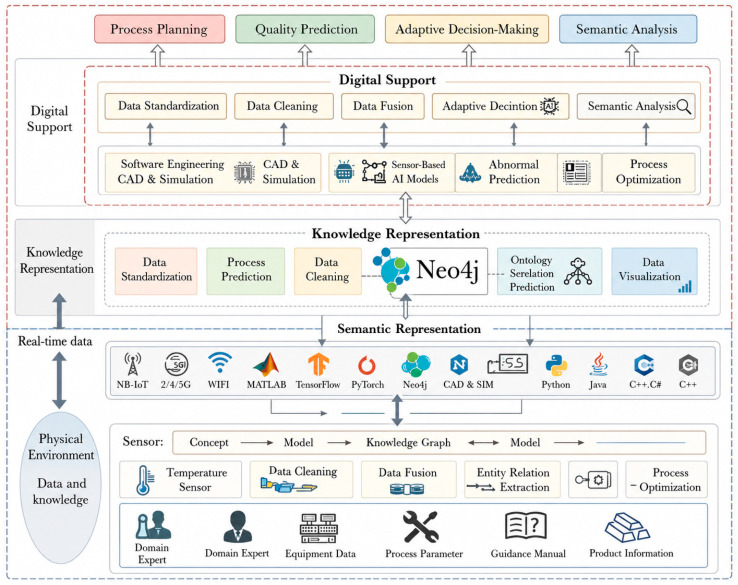
Knowledge representation and digital support in intelligent forging, showing the integration of knowledge sources, semantic representation, and digital tools for process planning, quality prediction, and adaptive decision-making. Arrows indicate data, knowledge, and control flow, and colored borders distinguish the principal architectural layers.

As shown in Figure 7, knowledge representation and digital support should be understood as a connected architecture. Rules and cases are first formalized as entities and relations; these relations are then linked with real-time data, FEM results, inspection records, and digital-twin modules. In practical terms, this architecture can support queries such as which reheating strategy is suitable for a titanium-alloy forging with a narrow forming window, which preform family has reduced folding risk for a similar closed-die part, or which historical cases show abnormal distortion after machining. In this sense, knowledge representation improves interpretability and decision continuity, whereas digital support provides the operational basis for knowledge reuse and closed-loop regulation.

## 4. Intelligent Process Design, Prediction, and Control

### 4.1. Process and Die/Preform Optimization

Intelligent process design in forging should be interpreted as a constrained engineering optimization problem rather than a generic AI task. The process route, billet geometry, heating schedule, preform sequence, die cavity, lubrication condition, and press parameters must satisfy material flow, equipment capacity, defect suppression, microstructure control, die life, and cost constraints simultaneously [2,12,14,15]. For closed-die forging, this usually means avoiding underfill, flash instability, laps, excessive load, and die over-stress. For large open-die forging, the key objectives are more likely to be center consolidation, deformation penetration, temperature uniformity, and microstructure/property homogeneity. Therefore, intelligent process design must be evaluated against process-specific quality targets.

Among these tasks, die and preform optimization are particularly important because they directly affect metal flow, cavity filling, forming load, defect occurrence, and die life. Existing work has shown that numerical optimization can improve deformation uniformity and dimensional accuracy, while machine-learning-assisted design can further accelerate the generation of feasible preform candidates [2,12,14,15,42]. Therefore, process optimization in intelligent forging is no longer limited to a single target, such as minimizing forging load or avoiding underfill, but is increasingly treated as a multi-objective problem involving defect suppression, die-stress control, dimensional precision, and process robustness.

**Figure 8:**
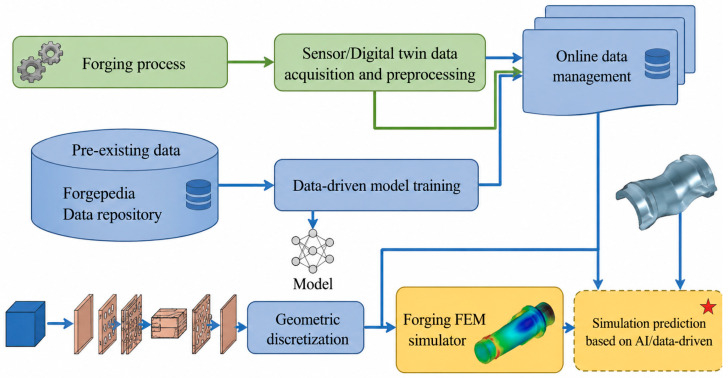
Representative workflow of AI-assisted preform design for forging, adapted from Ref. [42]. Arrows indicate the workflow and data flow. The star marks the AI/data-driven simulation-prediction output.

A representative example is the CNN-based preform design method proposed by Lee et al. [42], in which the final forging geometry is transformed into a digital representation and used to generate candidate preforms. As illustrated in Figure 8, this workflow demonstrates how AI can accelerate the early design stage, but it should still be treated as an AI-assisted design tool rather than an autonomous solution. Candidate preforms must be checked by FEM, feasibility rules, die-stress constraints, and engineering review before production. This distinction is important because preform-design models may perform well for geometries similar to the training database but fail when material flow, friction, flash design, or equipment constraints differ substantially.

### 4.2. Microstructure and Property Prediction

Microstructure and property prediction is a central task in intelligent forging because the performance of forged components depends not only on geometric accuracy, but also on the evolution of grain structure, phase constitution, texture, precipitation state, and the resulting mechanical properties. In hot forging, these responses are governed by coupled thermal and deformation histories, making process–structure–property relationships highly nonlinear and strongly path dependent [43,44,45,46,47]. Consequently, reliable prediction requires modeling strategies that can account for both metallurgical mechanisms and process variability.

Conventional approaches have mainly relied on phenomenological, physically based, or mesoscale models to describe recrystallization, grain growth, and flow behavior [43,44]. These models remain essential because they provide a physically interpretable framework for understanding how strain, strain rate, and temperature influence microstructural evolution during forging. However, their industrial application is often limited by the difficulty of parameter calibration, especially for large forgings and multi-stage processes. For this reason, recent research has increasingly combined conventional microstructure modeling with machine learning and reduced-order strategies to improve prediction efficiency and industrial applicability [45,46,47].

**Figure 9:**
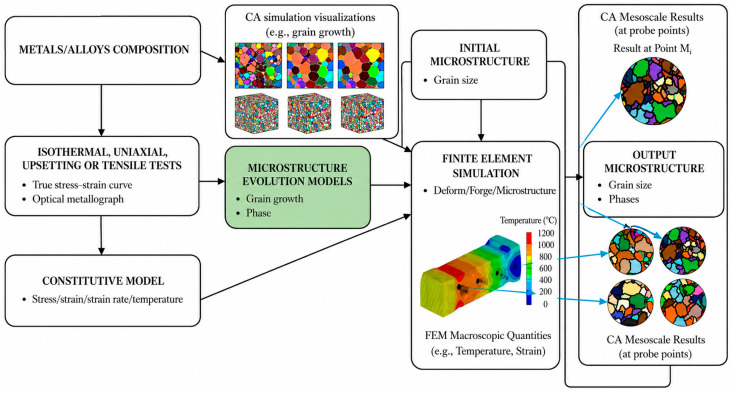
Representative modeling routes for microstructural evolution and property prediction in hot forging, adapted from Ref. [43]. Arrows indicate the modeling and information-flow routes.

As illustrated in Figure 9, current modeling routes for microstructural evolution and property prediction are becoming progressively more integrated. Mechanism-based models remain necessary for interpreting the effects of temperature, strain, strain rate, dynamic recrystallization, grain growth, phase transformation, and precipitation [43,44]. Data-driven methods can improve prediction efficiency when sufficient process and inspection data are available [45,46,47]. However, validation is critical: a microstructure model should be tested not only against the training dataset but also against independent forging trials, different locations within the same forging, new process windows, or separate alloy batches. Without such validation, high prediction accuracy may simply reflect interpolation within a narrow dataset rather than real process understanding.

### 4.3. Online Prediction, Decision-Making, and Closed-Loop Control

Online prediction and decision-making are essential for moving forging from offline optimization toward adaptive manufacturing. In industrial production, process disturbances may arise from billet temperature fluctuation, friction variation, die wear, equipment deviation, and material inconsistency. Under such conditions, fixed process parameters are often insufficient to ensure stable quality. For this reason, intelligent forging increasingly emphasizes real-time prediction of process states and product responses, followed by decision-making and control actions that compensate for observed or predicted deviations [38,47,48,50].

Although closed-loop control in metal forming has been studied for more than a decade, its implementation remains challenging because many relevant product properties are not directly observable during forming [48,50]. In forging, online prediction therefore plays a key bridging role: it converts measurable variables, such as temperature, force, displacement, or surface signals, into estimates of less accessible states, including local strain history, microstructural evolution, defect risk, and property trends. Once these predicted states are available, they can be used to update process windows, adjust stroke sequences, modify waiting times, or trigger process interventions.

**Figure 10:**
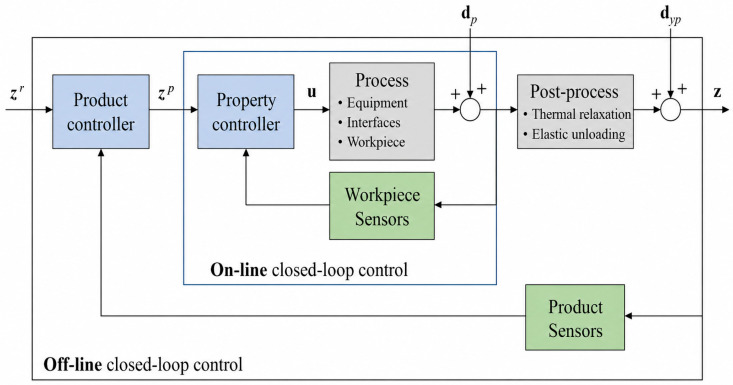
General framework of online prediction and closed-loop control in metal forming, adapted from Ref. [48]. Arrows indicate feedback and control flow in the online and offline closed-loop paths.

As shown in Figure 10, online prediction, decision-making, and closed-loop control should be treated as a continuous control chain. Lu and Huang [38] proposed a two-level modeling framework for intelligent integration control in time-varying forging processes, and DeepForge further showed that model predictive control can be linked with learning-based microstructure prediction [47]. Nevertheless, online control in forging is more difficult than offline prediction because key quality variables, such as internal strain, recrystallized fraction, residual stress, and final mechanical properties, are not directly observable during deformation. A practical closed-loop system therefore needs state estimation, uncertainty evaluation, intervention rules, and safe fallback strategies rather than only a high-accuracy prediction model.

### 4.4. Digital Twin and Adaptive Regulation

Digital twin technology provides an advanced framework for integrating process simulation, real-time data, and decision support in intelligent forging. Unlike conventional digital models, a digital twin is characterized by continuous interaction between the physical process and its virtual counterpart. This makes it particularly suitable for forging, where process states evolve dynamically and where offline models alone are often insufficient to support adaptive regulation [17,24,36,38,51].

In forging-related applications, digital twins can support several key functions. They can synchronize simulation-based knowledge with online measurements to improve the prediction of process states and quality responses, enable virtual testing and scenario analysis before physical intervention, and support adaptive regulation by updating model states and decision rules in response to incoming process information [17,24,36,51]. Therefore, the value of digital twins in intelligent forging lies not only in visualization, but also in prediction, optimization, and feedback-driven regulation.

**Figure 11:**
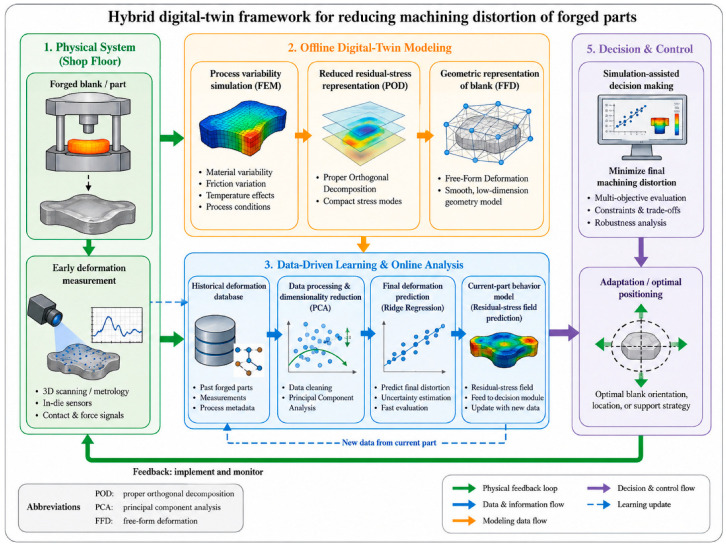
Closed-loop hybrid digital-twin framework integrating offline modeling, data-driven online learning and adaptive control for reducing machining distortion of forged parts.

The hybrid digital twin proposed by Chabeauti et al. [24] is discussed here as the single illustrative case of digital-twin-enabled distortion prediction for forged parts. In this framework, thermomechanical simulation, online measurements, and machine learning are combined to predict distortion during subsequent machining, demonstrating how reduced-order physics, real-time data, and adaptive prediction can be integrated into a practical decision-support system. As illustrated in Figure 11, the broader implication is that digital twins become most valuable when they connect process data, predictive models, and engineering knowledge within a unified feedback loop for adaptive regulation.

## 5. Industrial Applications and Current Challenges

### 5.1. Representative Industrial Applications

The industrial relevance of intelligent forging is most evident when it is linked to quality-critical production scenarios rather than presented as a general digitalization trend. In practical applications, intelligent methods are being extended from offline simulation to preform design, process-window optimization, microstructure/property prediction, distortion control, and adaptive decision support [2,24,38,39,46,47]. These applications are particularly important for aerospace, energy, transportation, and heavy-equipment forgings, where trial-and-error is expensive and where geometry, microstructure, residual stress, dimensional stability, and mechanical properties must be controlled simultaneously.

Current industrial applications can be broadly grouped into three categories. The first is design-oriented application, in which AI- or optimization-assisted methods are used to improve process routes, die design, and preform generation before production. The second is prediction-oriented application, in which simulation, data-driven models, or hybrid approaches are employed to predict geometry, microstructure, or mechanical properties during or after forging. The third is control-oriented application, in which online measurements are combined with predictive models to support process adjustment, adaptive machining, or feedback regulation [2,24,38,46,47]. Although these application routes differ in maturity and implementation depth, they all reflect the same overall trend: forging is evolving from experience-based planning toward intelligent, data-connected, and decision-supported manufacturing.

At the application level, the key implication of the studies discussed above is that industrial value arises from integration rather than from repeatedly listing individual case examples. Design-oriented tools can shorten die and preform development cycles, prediction-oriented tools can connect process records with microstructure and property estimates, and control-oriented tools can use sensing and model updates to reduce quality variation. Therefore, the maturity of intelligent-forging applications should be evaluated by cross-stage connectivity, validation under production variability, and compatibility with existing shop-floor systems.

### 5.2. Comparison of Current Technical Routes

Although the term “intelligent forging” is now widely used, current technical routes differ substantially in their objectives, data requirements, validation methods, and deployment maturity. Four routes can be identified: (i) simulation-driven design based on FEM and mechanism models; (ii) data-driven prediction using machine learning and industrial datasets; (iii) hybrid predictive control combining physical models, online sensing, and learning; and (iv) digital-twin-driven regulation integrating physical assets, virtual models, data streams, and decision modules [2,17,36,38,39,46,47,48,50,51]. These routes should be evaluated not only by prediction accuracy but also by physical consistency, validation depth, transferability, real-time feasibility, and engineer trust.

To avoid treating these technical routes as equivalent in maturity, Table 3 compares their typical applications, current maturity, required validation, key barriers, and representative references. The comparison shows that FEM-based simulation and conventional preform optimization are relatively mature, whereas microstructure/property prediction, hybrid reduced-order twins, knowledge-graph-assisted planning, and large-model-assisted forging still require stronger cross-batch validation, interface standardization, online robustness testing, and long-term industrial maintenance.

These routes should not be viewed as mutually exclusive. A realistic intelligent-forging system will usually combine them: FEM provides mechanism-consistent training data and process understanding; data-driven models accelerate prediction; hybrid models update states under production disturbance; knowledge graphs preserve rules and cases; and digital twins organize these elements within a virtual-physical feedback loop. The critical issue is therefore not which algorithm is “most intelligent”, but whether the integrated route can solve a specified forging problem under validated industrial conditions.

As summarized in Table 3, the main difference among current technical routes lies in their validation depth and deployment maturity rather than in the nominal algorithm type. Simulation-driven methods emphasize process understanding and offline verification; data-driven methods emphasize rapid response but require independent validation; hybrid methods emphasize predictive robustness under disturbance; and digital-twin-driven routes emphasize system-level integration. This text-based comparison replaces the previous schematic to avoid redundancy and to keep the maturity assessment directly linked to the tabulated evidence.

### 5.3. Key Bottlenecks in Data Quality, Model Credibility, and Deployment

Despite the progress outlined above, several bottlenecks continue to limit deployment. The first is data quality. Forging data are often incomplete, asynchronous, and weakly labeled. Variables such as local strain history, internal temperature, recrystallized fraction, and bulk residual stress are difficult to measure online, while offline microstructure and property labels are sparse and delayed [17,36,39]. As a result, a model trained on available signals may predict apparent correlations rather than causal process–structure–property relationships. Data governance must therefore include timestamp synchronization, sensor calibration, metadata recording, abnormal-data filtering, and traceable linkage between process histories and final quality results.

The second bottleneck is model credibility. A useful intelligent-forging model must be accurate, physically plausible, stable under disturbance, and reliable outside the exact training domain. Real-condition validation should include independent forging batches, new geometries, different alloy grades or heat-treatment states, altered equipment or die conditions, sensor-noise disturbance, comparison with calibrated FEM simulations, and verification using offline dimensions, defects, grain size, residual stress, or mechanical properties. Reporting only random train-test accuracy is insufficient for industrial deployment because production drift, material-batch variation, and sensor degradation can invalidate a model that appears accurate on historical data.

Operationally, model validation in intelligent forging should be reported at several distinct levels: numerical verification against calibrated FEM; independent laboratory forging experiments; verification using industrial batches; extrapolation to new geometries, dies, or forming routes; transfer to different alloys, heat-treatment states, or material batches; robustness testing under sensor noise, missing signals, and synchronization errors; and long-term monitoring under production drift. This hierarchy makes validation more actionable because each level answers a different question about whether a model is physically correct, experimentally reliable, industrially transferable, and robust during shop-floor deployment.

The third bottleneck lies in deployment and system integration. Forging workshops often involve heterogeneous presses, furnaces, manipulators, legacy control systems, manual decisions, and fragmented data infrastructures [17,25,26,36,51]. Digital twins, knowledge graphs, and integrated management platforms can support system-level deployment only when they connect data streams, model updates, process rules, and decision logic in a maintainable way. For high-value components, engineers must be able to understand why a recommendation is given, what uncertainty is involved, and what evidence supports the action. Thus, deployment trust depends on interpretability, safety constraints, and human-in-the-loop review as much as on algorithmic performance.

**Figure 12:**
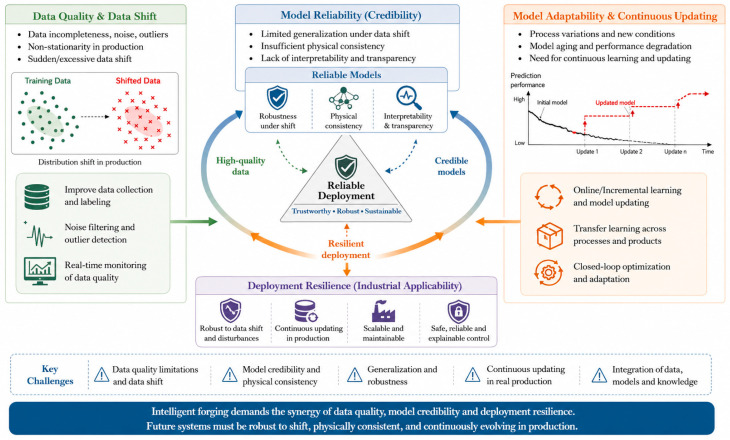
Schematic illustration of the relationship among data quality, model robustness, and reliable deployment in intelligent manufacturing, adapted from Ref. [52]. Arrows indicate the influence and feedback relationships among data quality, model reliability, and deployment resilience.

As illustrated in Figure 12, the main challenges of intelligent forging are not purely algorithmic. Data quality determines the observability of process states, model credibility determines whether predictions can be trusted under changing conditions, and deployment resilience determines whether the system remains operable over long production cycles. Future studies should therefore report not only accuracy but also validation setting, data source, transferability, uncertainty, failure cases, and the role of engineering supervision.

## 6. Future Perspectives and Conclusions

### 6.1. Large Models and Human-in-the-Loop Intelligent Forging

The future development of intelligent forging will likely be influenced by large models and human-in-the-loop decision architectures, but their role should be defined realistically. Large models may be useful for multimodal document understanding, technical information retrieval, historical-case recovery, diagnostic explanation, process-card interpretation, and supervised recommendation. These tasks are valuable because forging knowledge is dispersed across papers, standards, process cards, simulation reports, inspection records, and expert experience.

However, large models should not be presented as a near-term substitute for validated process models or expert decision-making. Forging is governed by strong thermomechanical coupling, path-dependent microstructure evolution, sparse industrial data, and safety-critical quality requirements. Therefore, short-term applications should remain retrieval-, diagnosis-, and recommendation-oriented. By contrast, real-time autonomous modification of forging parameters without human review, physical constraints, or plant-specific validation should be regarded as a speculative long-term objective rather than a mature industrial capability.

Accordingly, this review treats large-model-assisted forging as a supporting layer rather than as a validated control architecture. Its near-term roles are limited to literature and standard retrieval, process-card interpretation, historical-case recovery, diagnostic explanation, anomaly triage, and engineer-supervised recommendation. Any use of such models for parameter adjustment should be filtered through validated mechanism-based models, plant-specific rules, uncertainty thresholds, safety constraints, and responsible engineer approval. This more cautious description avoids implying a technological maturity that has not yet been demonstrated in industrial forging.

### 6.2. Standardization and Trustworthy Deployment

Despite the rapid progress of intelligent forging, its large-scale industrial adoption is still constrained by insufficient standardization and limited deployment trustworthiness. Standardization is particularly important because intelligent forging involves heterogeneous data, multiple software and hardware platforms, and strongly coupled process–material–quality relationships. At present, fragmented data formats, inconsistent definitions of process variables, nonuniform quality labels, and weak model-interface specifications still hinder data reuse, model transfer, and system integration across different forging lines, equipment platforms, alloys, and workshops.

Therefore, standardization should be regarded as a core research and engineering task rather than an auxiliary issue. Future standard systems for intelligent forging should cover process-variable definitions, sensor metadata, timestamps, workpiece identifiers, deformation-history records, heat-treatment parameters, inspection labels, microstructure descriptors, process-knowledge ontologies, model interfaces, validation protocols, and model-update mechanisms. These elements are essential for connecting sensing systems, finite-element simulations, machine-learning models, knowledge bases, digital twins, and production-management platforms within a stable and traceable industrial environment. Only with unified data semantics and validation rules can results obtained from different equipment, alloys, production batches, or research studies be reliably compared, reproduced, and reused.

In addition to standardization, trustworthy deployment is another prerequisite for industrial implementation. Intelligent forging models must be robust to data shifts, sensor noise, changing boundary conditions, alloy variations, and equipment differences. They should also maintain physical plausibility, provide interpretable outputs for engineers, support cybersecurity-aware data management, and remain maintainable during long-term production. Accordingly, future research should move beyond accuracy-oriented black-box prediction and place greater emphasis on resilient, transparent, validated, and human-supervised intelligent-forging systems. This shift is essential for transforming intelligent forging from laboratory-level demonstrations into reliable industrial applications.

### 6.3. Conclusions

This review clarifies intelligent forging as a mechanism–data–knowledge fusion paradigm for full-chain process perception, modeling, prediction, decision-making, and adaptive regulation. The revised manuscript emphasizes that intelligent forging must be grounded in real forging routes, alloy systems, quality objectives, and validation settings rather than discussed only as a general intelligent-manufacturing concept.

Mechanism-based models, data-driven models, and knowledge representations play complementary roles. Mechanism models ensure physical plausibility, data-driven models improve response speed and nonlinear mapping capability, and structured knowledge preserves engineering rules, expert experience, and decision traceability. Hybrid models are most useful when their type and application are clearly specified, such as FEM-trained surrogates for preform design, physics-informed models for microstructure prediction, reduced-order twins for distortion prediction, sensor-updated models for online quality estimation, and expert-rule-integrated models for process planning.

From the viewpoint of maturity, FEM-based process simulation, conventional process-window analysis, and die/preform optimization are the most consolidated applications. Microstructure and property prediction is moving toward maturity when it is supported by calibrated thermomechanical histories, alloy-specific experiments, and independent validation. Digital twins, sensor-updated hybrid models, and adaptive control are highly promising but remain under validation because they require stable sensing, model updating, uncertainty handling, and in-plant verification.

The critical barriers are reliable sensors, synchronized and standardized data, sparse microstructure/property labels, model transferability between equipment and materials, credible validation under real production disturbance, long-term maintainability, and engineers’ trust. Future studies should report databases or data sources, process/material details, input and output variables, validation methods, limitations, and maturity level so that intelligent forging can move from conceptual frameworks to deployable industrial systems.

Overall, intelligent forging should evolve from isolated predictive tools toward integrated, explainable, validated, and human-supervised manufacturing systems for high-performance forged components. The most realistic near-term pathway is not full autonomy, but a trustworthy framework in which sensors, mechanism models, data-driven prediction, knowledge reasoning, and expert review work together to improve quality, robustness, and decision efficiency.

## Figures and Tables

**Table 1:** Representative technical advances in intelligent forging.

Institution/Company	Main Focus	Representative Progress
SMS Group	Digital process design; intelligent line control	Automated forging systems
Hitachi Metals	Process–performance mapping; microstructure prediction	DL-based microstructure prediction
Bharat Forge Kilsta	Intelligent temperature control; energy optimization	AI-based thermal control
Airbus–Siemens	Digital thread; quality traceability	Lifecycle quality management
Chongqing University	Multi-field modeling; quality prediction; digital twins	Intelligent regulation framework
Wanhang Die Forging	Precision forming; process data acquisition; smart production	Intelligent die-forging line
Beijing Research Institute of Mechanical & Electrical Technology	Forging–heat coordination; data acquisition; traceability	Integrated cloud-control platform
Central South University	Intelligent control of process and microstructure/property evolution	Closed-loop optimization methods

**Table 2:** Main categories of hybrid models for intelligent forging, their process-specific applications, and representative supporting references.

Hybrid Model Type	Core Principle	Typical Forging Application	Validation Focus	Main Limitation	Representative Articles/References
FEM-trained surrogate model	Train a fast model using high-fidelity simulation data	Preform design, load prediction, die-stress evaluation, defect-risk screening	Comparison with FEM and selected experiments	Accuracy decreases outside the simulated design space	[30,31,32,33,42]
Physics-informed learning model	Embed physical constraints, constitutive relations, or kinetics into learning	Microstructure/property prediction and sparse-data extrapolation	Independent experiments and physical-consistency checks	Requires correct physical assumptions and calibrated parameters	[16,17,18,19,43,44,45,46,47]
Reduced-order digital twin	Simplify a high-fidelity thermomechanical model for rapid updating	Distortion prediction, process-state mapping, adaptive regulation	Real part/process cases and online/offline comparison	Plant-specific calibration and maintenance are demanding	[20,21,22,23,24,48]
Sensor-updated model	Use online force, displacement, temperature, image, or acoustic signals to update states	Deviation detection, quality-risk estimation, process-window adjustment	Noise robustness and independent production batches	Limited by sensor reliability, synchronization, and drift	[17,18,20,36,48]
Expert-rule-integrated model	Combine process cards, empirical rules, and knowledge graphs with prediction	Process planning, reheating decisions, lubricant selection, die-wear alerts	Expert audit, case retrieval, and production feedback	Rule completeness and knowledge maintenance are difficult	[25,26,27,49]

**Table 3:** Maturity classification of major technical routes in intelligent forging with representative supporting references.

Technical Route	Typical Applications	Current Maturity	Required Validation	Key Barrier	Representative Articles/References
FEM and mechanism-based simulation	Die filling, load prediction, preform sequence, defect-risk analysis	Consolidated for offline design	Comparison with experiments, industrial trials, and sensitivity analysis	Computational cost and boundary-condition uncertainty	[2,3,9,24,38,39]
Conventional optimization and preform design	Die/preform optimization, process-window design, load reduction	Consolidated to semi-consolidated	FEM verification and selected physical trials	Generalization to complex or new geometries	[12,14,15,30,31,32,33,42]
Microstructure/property prediction	Grain size, recrystallization, mechanical-property mapping	Under maturation	Independent alloy batches, different locations, and process windows	Sparse labels and alloy-specific calibration	[16,17,36,39,41,45,46,47]
Hybrid models and reduced-order twins	Fast prediction, state updating, distortion prediction, adaptive regulation	Under validation	New geometries, sensor noise, production drift, and in-plant cases	Model updating, uncertainty, and maintainability	[17,20,21,22,23,24,36,40,41,48]
Knowledge graphs and expert-rule models	Process planning, case retrieval, defect diagnosis, decision traceability	Emerging	Expert audit, historical-case recovery, and production feedback	Knowledge completeness and semantic standardization	[25,26,27,49]
Large-model-assisted forging	Document retrieval, case recovery, diagnostic support, supervised recommendation	Prospective	Human review, safety constraints, and process-specific grounding	Risk of unsupported recommendations and lack of physical guarantee	[18,22,24,36,48,51,52]

## Data Availability

No new data were created or analyzed in this study. Data sharing is not applicable to this article.
